# Soft Water Level Sensors for Characterizing the Hydrological Behaviour of Agricultural Catchments

**DOI:** 10.3390/s110504656

**Published:** 2011-04-28

**Authors:** Armand Crabit, François Colin, Jean Stéphane Bailly, Hervé Ayroles, François Garnier

**Affiliations:** 1 Montpellier SupAgro, UMR LISAH/2 place Pierre Viala, Montpellier 34060, France; E-Mail: colinf@supagro.inra.fr; 2 AgroParisTech, UMR LISAH, UMR TETIS/2 place Pierre Viala, Montpellier 34060, France; E-Mail: jean-stephane.bailly@agroparitech.fr; 3 IMFT-CNRS, UMR5502/1 Allée du Professeur Camille Soula, Toulouse 31400, France; E-Mail: Herve.Ayroles@imft.fr; 4 INRA, UMR LISAH/2 place Pierre Viala, Montpellier, 34060 France; E-Mail: garnierf@supagro.inra.fr

**Keywords:** water stage recorder, capacitance, agricultural catchment behaviour, sensor application

## Abstract

An innovative soft water level sensor is proposed to characterize the hydrological behaviour of agricultural catchments by measuring rainfall and stream flows. This sensor works as a capacitor coupled with a capacitance to frequency converter and measures water level at an adjustable time step acquisition. It was designed to be handy, minimally invasive and optimized in terms of energy consumption and low-cost fabrication so as to multiply its use on several catchments under natural conditions. It was used as a stage recorder to measure water level dynamics in a channel during a runoff event and as a rain gauge to measure rainfall amount and intensity. Based on the Manning equation, a method allowed estimation of water discharge with a given uncertainty and hence runoff volume at an event or annual scale. The sensor was tested under controlled conditions in the laboratory and under real conditions in the field. Comparisons of the sensor to reference devices (tipping bucket rain gauge, hydrostatic pressure transmitter limnimeter, Venturi channels…) showed accurate results: rainfall intensities and dynamic responses were accurately reproduced and discharges were estimated with an uncertainty usually acceptable in hydrology. Hence, it was used to monitor eleven small agricultural catchments located in the Mediterranean region. Both catchment reactivity and water budget have been calculated. Dynamic response of the catchments has been studied at the event scale through the rising time determination and at the annual scale by calculating the frequency of occurrence of runoff events. It provided significant insight into catchment hydrological behaviour which could be useful for agricultural management perspectives involving pollutant transport, flooding event and global water balance.

## 1. Introduction

Agricultural activities, by modifying land use patterns, water flow path and pollutant leaching, strongly impact water balance and water quality at various spatial scales [1,2]. Land use modifications through agricultural practices, such as tillage, modify surface roughness and impact soil hydrological properties, including local surface runoff, infiltration and surface storage [3–5]. Man-made channels resulting from the constant efforts of farmers to adapt landscapes to the constraints of agricultural production influence water transfer from fields to catchment outlets [6,7]. Water quality is affected by the high leaching potential of herbicides [8,9] and, in particular, by transport processes taking place in surface water at both field and catchment scales [10,11].

Beyond these evidences, agricultural land management practices that are compatible with the preservation of water resources have to be defined. Hydrological diagnoses are needed in order to choose the alternative land uses, cultivation practices and/or their spatial arrangements [12–14].

Water resource managers who need to estimate and monitor water fluxes at the catchment scale have to face epistemic uncertainties resulting from scarce data. As reported in [15], many parts of the world are either ungauged or poorly gauged, *i.e.*, without rainfall and stream flow measurements. Even if water-monitoring networks exist [like those recommended by the Water Framework Directive (WFD; 2000/60/EC) in the European Union], they mostly concern large river catchment outlets at high time frequency. The lack of hydrological records at the elementary catchment scale upstream which is the key-scale to relate agricultural activities and impacts on water bodies, is obvious [16–18].

Facing the need for rainfall and runoff records, usual measurements involve rain gauge recorders (weighing precipitation gauges or tipping bucket rain gauges) and liminimetric sensors (hydrostatic pressure transmitters, air bubbling level transmitters) coupled with the building of weirs or calibrated channels, *i.e.*, Venturi channels, to estimate discharges. All these techniques are expensive, of high-power consumption, invasive and require heavy maintenance operations [19,20]. As a consequence, this usual hydrometric equipment is scarce in space but with high time frequencies. For more balanced space over time hydrological measurements, there is room and a crucial need to develop alternative soft metrologic approaches that permit one to estimate water fluxes in catchments with an higher spatial sampling rate [21,22]. Capacitive sensors may constitute such an alternative [23]. As reported in [24], they can be used to directly sense motion, chemical composition or electric fields, but also to indirectly sense pressure, acceleration, fluid levels, and fluid composition, which can be converted into motion or dielectric constants. Their use still remains unusual [25], especially to characterize hydrological variables. However, simple conditioning circuits, low cost and stability of the technology are attractive advantages.

The aim of this paper is to propose a hydrological spatio-temporal sampling design for elementary cultivated catchments based on an innovative handy, low-cost, low power/energy consumption, non-invasive and robust water level sensor. The sensor was used to monitor water-levels in order to estimate both the rainfall and the stream flow characteristics of several elementary agricultural catchments. The sensor and the experimental designs are presented. The results of tests performed in the laboratory, under controlled conditions and under field conditions are discussed. Finally, the proposed experimental strategy was applied to characterise the hydrological behaviour of eleven Mediterranean catchments in term of runoff response frequency, lag-time and water budget.

## 2. Materials and Methods

### 2.1. Sensor Design

#### 2.1.1. Capacitive Sensor

**Figure 1.**
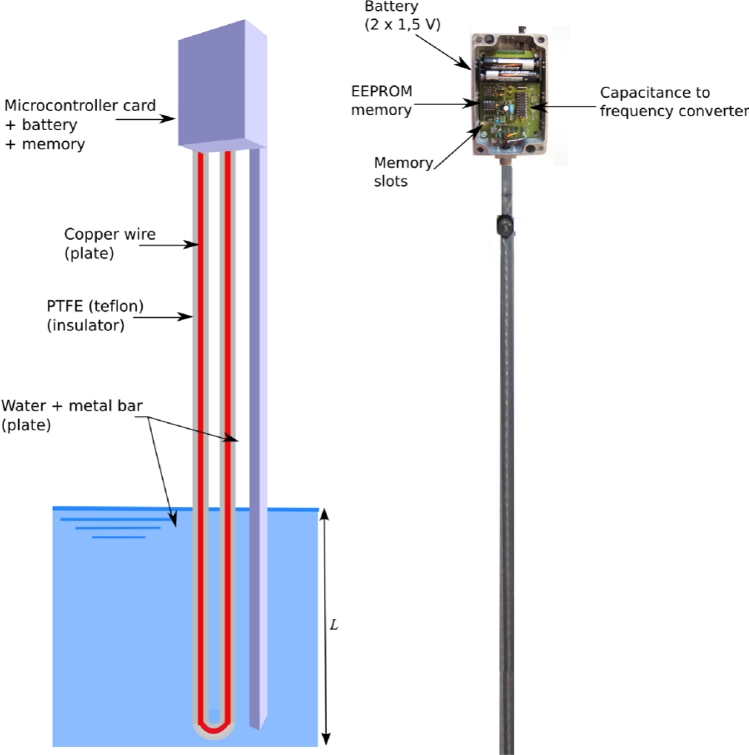
Sensor scheme.

The Institute of Fluid Mechanic of Toulouse (IMFT) initiated the development of a limnimetric sensor that works as a capacitor, usually constituted by two plates and an insulator. In this study, the sensor is constituted by four main elements (Figure 1): (i) a copper wire which forms one plate of the capacitor that is wrapped in (ii) a polytetrafluoroethylene (PTFE)-insulated wire, the dielectric, which is fixed to a stainless steel rod, (iii) water that forms the other plate of the capacitor and determines the length *L* of the capacitor and (iv) the data logger, composed of the micro-controller card and two usual batteries (AAA 1,5 V), which is housed in a waterproof container.

Considering that the sensor capacitance will vary with the thickness and dielectric constant of the insulation, it is necessary to use a stable material, such as PTFE, which does not absorb water. The capacitance is calculated in function of the geometry of the conductors and the dielectric properties of the insulator between the conductors. For a cylindrical capacitor the capacitance expression is:

(1) $$C_{\text{sensor}}\;=\;\frac{2\pi \;\times \;{ɛ}_{0}\;\times \;{ɛ}_{r}\;\times \;L}{\text{ln}\left(\frac{R_{1}}{R_{2}}\right)}$$

where *C_sensor_* is the capacitance of the sensor (F), *ɛ_0_* an electric constant (*ɛ_0_* ≈ 8.854 × 10^−12^ pF.m^−1^), *ɛ_r_* is the relative static permittivity (sometimes called the dielectric constant) of the material between the plates (*ɛ_r_* ≈ 2.1 for the Teflon), *L* the length of the capacitor (m) and *R_1_* and *R_2_*, the radii of the inner and outer cylinders, respectively. Capacitance is proportional to the plate area and therefore works as a spacing-variation capacitor. π, *ɛ_0_*, *ɛ_r_*, *R_1_*, *R_2_*, being constants, the capacitance is linearly related to *L* and consequently to the water level. For *L* equal to 0 m the capacitance was 903.6 pF and for *L* equal to 1.20 m the capacitance was 7,665 pF. Hence, the sensor could be used as a water level sensor where the higher the water level is, the greater the capacitance. A digital signal was required for easy interfacing to a computer. This requirement was met by coupling the capacitor with a capacitance to frequency converter (LMC 555 CMOS timer).

The converter produces a frequency output which is linear with water level. Used in astable mode operation, charge, discharge time, period and frequency of the signal could be calculated as follows:

(2) $$t_{1}\;=\;0.693(R_{a}\;+\;R_{b})\;C_{\text{tot}}$$

(3) $$t_{2}\;=\;0,\;693(R_{b})\;C_{\text{tot}}$$

with:

(4) $$C_{\text{tot}}\;=\;C_{\text{sensor}}\;+\;C_{\text{converter}}$$

and:

(5) $$T\;=\;t_{1}\;+\;t_{2}\;=\;0.693(R_{a}\;+\;2{\text{R}}_{b})\;C_{\text{tot}}$$

(6) $$f\;=\;\frac{1}{T}\;=\;\frac{1.44}{(R_{a}\;+\;2{\text{R}}_{b})C_{\text{tot}}}$$

where *t_1_* is the charge time (output high), *t_2_* the discharge time (output low), *R_a_* and *R_b_* resistors (=47 kΩ), *C_converter_* the capacitance of the converter, which is constant (=100 pF), *C_tot_* the total capacitance, *T* the period of the signal and *f* the frequency of oscillation of the signal.

As shown by Equation (5)*T* and *f* are function of the total capacitance that varies according to *C_sensor_* and hence to the water level. For *L* equal to 0 m the frequency was 11,325 Hz and for *L* equal to 1.20 m the frequency was 1,335 Hz. Then, the water level was estimated by counting the number of signals over 1/16 s during an adjustable period (1 min up to 1 day). The higher the water level is, the lower the frequency. Output frequency is then recorded using two Electrically Erasable Programmable Read-only Memory (EEPROM) units with a 32 KB memory capacity each, which allows us to realize 32,000 measurements. Full memory is reached after three weeks of measurements when the data acquisition step is set at one minute. Sensor memory could be extended to 128 KB by adding two more EEPROM memory on the available slots.

#### 2.1.2. Data Logger

A low consumption Microchip PIC16LF628 micro-controller with firmware written in Pascal (mikroPascal) controls the data logger. To ensure a better battery life by low energy consumption, the micro controller remains in a sleep mode but automatically wakes up every 4 s for a very short time. During this short time, the clock verifies if a measurement is required: in that case, the measurement is performed and stored on EEPROM memory in the previously acquired data queue. At the same time, the micro-controller reads the binary state of a pin. When this state is 0, corresponding to a connection of the data logger to a communication wire, the micro-controller is maintained awake (in a normal mode) in order get ready to communicate with a PC.

**Figure 2.**
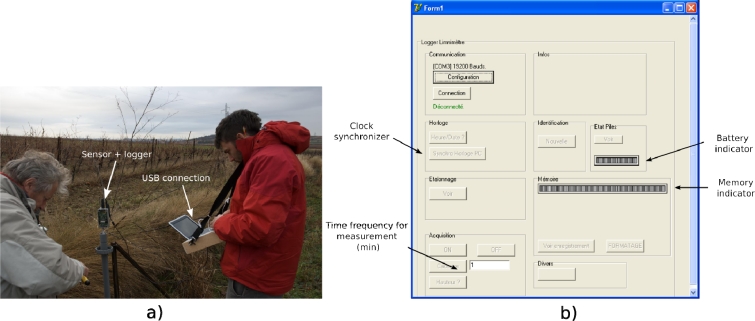
**(a)** Water level sensor used as a stage recorder at field. **(b)** Software ‘levelmeter’ interface.

Concerning the sensor device control by operators with a PC, a specific software, ‘Levelmeter’, has been developed in the Delphi language. This software allows one to configure the logger, to synchronize the sensor and PC clocks, to define the time frequency for measurements in periods ranging from 1 to 10 min, to define the linear law between frequencies and levels and to upload the data from the memory. It also permits visualization of the acquired data loaded in the memory [Figure 2(a,b)]. Communication between sensor and PC is performed through a wire with RS232 protocol without handshaking (TX,RX,GND). Digital data transfer speed is fixed to 19,200 baud. This results in a 68 seconds duration to download the total memory capacity.

#### 2.1.3. Monitoring Setting

The proposed monitoring setting was designed to be as handy, discrete and easy to install as possible, compared to usual hydrological devices, so building a small, lightweight device, which could provide efficient and high temporal resolution rainfall-runoff measurements was investigated in this study.

**Figure 3.**
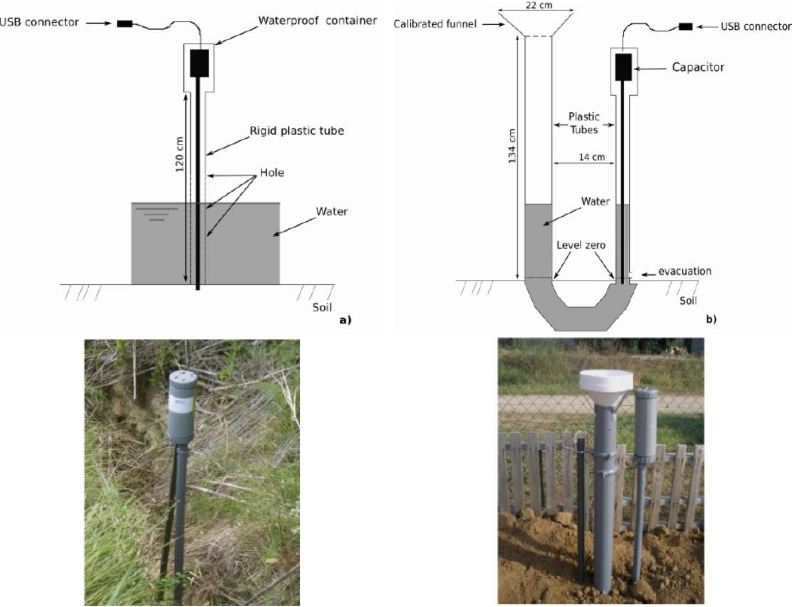
Monitoring setting scheme: (**a**) stage recorder, (**b**) rain gauge.

Considering these criteria, the monitoring setting was composed of a stage recorder coupled with a rain gauge using the capacitive sensor described above. Figure 3 shows the functional scheme. First, the sensor was used as a stage recorder installed in a channel. The sensor was installed into a break-through rigid plastic tube of 15 cm diameter [Figure 3(a)] in order to protect the PTFE wire from vegetative debris (leaves, piece of woods) and cobblestones that are frequently carried downstream when flow occurs in agricultural catchments. Secondly, the sensor was also used as a rain gauge [Figure 3(b)], built with a 22 cm diameter collection funnel, two connected vessels of 1 m high with the water level sensor installed in one of the two vessels. Based on the connected vessel principle, rainfall is collected through the calibrated funnel in the first tube. Water level is measured in the second vessel at an adjustable time step acquisition ranging from one minute up to few hours or days.

The uses of such a proposed monitoring setting are numerous: it can be built quite cheaply and consequently can be densely distributed in space; it is a nomad sensor in the sense that is easy and quick to install and uninstall in the field; due to its simple battery system, it does not need any connection to a high energy source and can be installed everywhere; due to its small size, it is a non-invasive and unnoticeable sensor which protects it from vandalism. Of course, to balance these advantages, it is expected to be less accurate than conventional sensors. The cost of the components is approximately 80 € and 100 €, respectively. The logger has a maximal temporal resolution of one minute that allows for characterizing, at a given location, rainfall-runoff events such as water level dynamics, events duration, rainfall intensity, rainfall amount (at event or annual scale) or high flow and low-flow periods. Furthermore, other variables of interest such as the water discharge could be estimated too.

### 2.2. Water Level to Water Discharge Conversion

To transform a water level to water discharge, there are four practical ways: (i) to measure the flow velocity at the same time as water level, (ii) to use some particular hydraulically controlled section (iii), to use pre-calibrating rating-curves and (iv) to use the mean empirical Gauckler-Manning-Strickler law [26], simply denoted hereinafter as Manning’s equation. The three first ways need additional heavy sensors or equipments and, long-term studies under flow conditions.

In a given cross-section of a channel, the water level can be converted to water discharge using the widely used Manning equation. The Manning equation was developed empirically in laboratory channels with optimal draining conditions and uniform flow and no downstream influences must be assumed to apply the equation. However, referring to [27] “…lacking a better solution, it is assumed that the equation is also valid for non-uniform reaches that are invariably encountered in natural channels if the energy gradient is modified to reflect only the losses due to boundary friction [28]”.

The instantaneous flow velocity (*v* [m.s^−1^]) and discharge *Q* [m^3^.s^−1^] in a channel are estimated as follows:

(7) $$v\;=\;\frac{1}{n}\;\cdot \;R_{h}^{2/3}\;\cdot \;\sqrt{S}$$

(8) Q = v ⋅ A

where *S* [dimensionless] denotes the slope of the water surface, which can be approximated by the slope of the channel bed; *A* [m^2^] denotes the cross-sectional area; *R_h_* [m] is the hydraulic radius equals to the ratio between A and *P,* the wetted perimeter [m]; *n* [s.m^−1/3^] denotes the Manning roughness coefficient. The channel’s slope and cross-section shape being temporally stable, it can be both estimated from topographic ground survey. The hydraulic radius depends on water level which is measured by the stage recorder. The term *n* can be estimated by choosing, in the abundant literature, the most suitable coefficient [29,30], according to cross-section characteristics (dimensions and shape), bed substrate and cover types (vegetation, concrete, plastic, *etc.*).

Assuming that Manning’s equation is relevant, the uncertainty in discharge estimation comes mainly from the uncertainty around *n*, *i.e.*, possible values for this parameter. Indeed, vegetation is a primary factor in the increase of the roughness and resistance in channels [31,32]. Hence, a maximum value should be used during periods of low flows and high vegetation density, and minimum values during high flows and low vegetation density. Therefore, discharge estimation is associated to an envelope curve which corresponds to an upper and a lower acceptable limits on discharge estimation. Hence, when the water level sensor is installed at a catchment outlet, discharge could be estimated with a given uncertainty and several indicators could be derived to characterize catchment hydrological behaviour.

### 2.3. Hydrological Indicators

#### 2.3.1. Catchment Dynamic Responses

Time series obtained using rainfall and stage recorders allowed separating the flood events. The separation was done according three steps [33]: (i) screening of the peak stages, (ii) identifying the starting time of the corresponding rainfall event and (iii) identifying the ending time of the rainfall event, which correspond to the ending time of the runoff event and when the discharge is equal to zero. Separating events allowed us to calculate the frequency of occurrence of a runoff event: *B*, defined as the frequency of rainfall event that did generate runoff on a given catchment (we chose to define a rainfall event if the amount was higher than the threshold equals to 5 mm). B is calculated as follows:

(9) $$B=\frac{\text{Number}\;\text{of}\;\text{events}\;\text{not}\;\text{producing}\;\text{runoff}}{\text{Total}\;\text{number}\;\text{of}\;\text{events}}$$

At the rainfall event scale, the rising time was calculated as the time from the beginning to the peak of the flood at the catchment outlet. This variable informs on the time of reaction of a catchment to a rainfall event: the shorter the rising time, the more sudden the catchment response. The rising time is the time interval between the start and the peak of a runoff event.

Considering the catchment hydrological behaviour, the main issues concerned temporal analysis of the intermittent outflows in the pollutant transport and the flood management perspectives and estimation of the water budget at a catchment scale from the global water resource management perspective.

#### 2.3.2. Water Budget at the Catchment Scale

Access to good-quality water-balance data at the local to global-scales is one of the most important prerequisites for sustainable development planning [34]. The water balance equation for a given time period is:

(10) R = Q + AET + DL + ΔS

where *R* (mm) is the amount rainfall, *Q* (mm) denotes the catchment runoff, *AET* (mm) denotes the actual evapotranspiration, *DL* (mm) denotes the deep drainage and *ΔS* (mm) denotes the variation of the storage in soil and groundwater.

On the annual scale, it is possible to assume that *ΔS* is non significant compared to the other terms. Moreover, considering the lithological configuration of the catchments and specificity of the Mediterranean climate and main hydrological processes diagnosed (*“Hortonian overland flow”*), we assume in a first step that the deep drainage was also non significant compared with the other terms. The equation was therefore simplified as follows:

(11) R = Q + AET

By measuring rainfall depth and estimating outflows through stage records and rating curves based on the Manning equation, the proposed experimental strategy permitted us to calculate two terms of the water balance on the eleven studied catchments. The third term (AET) was deduced from Equation (11).

### 2.3. Study Area

**Figure 4.**
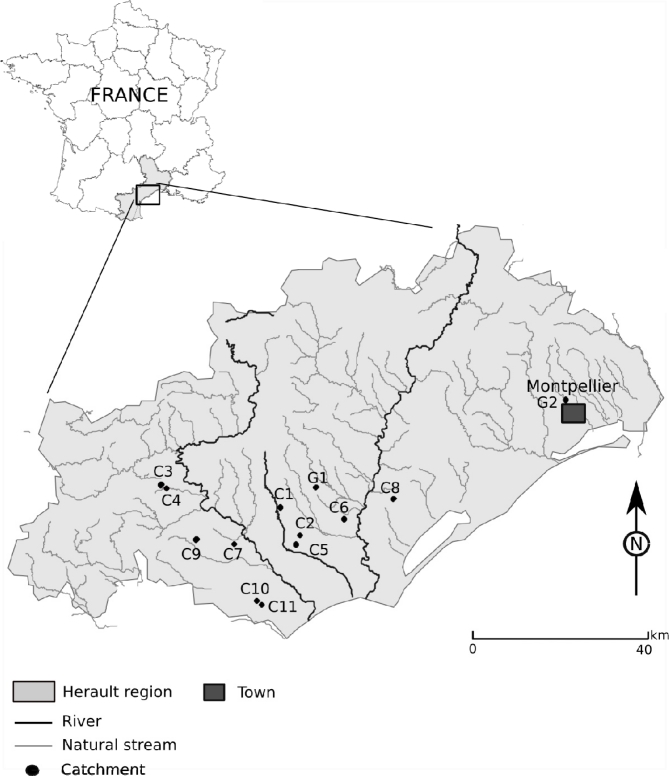
Catchment location.

The soft metrologic approach was proven in a real field application case. On the basis of the use of the water level sensors as rainfall and stream flow recorders, the indicators mentioned above were calculated in order to compare a set of catchments. Eleven small headwater catchments located in southern France were monitored during the hydrological year 2008–2009 at one minute time-intervals. The location of the catchments is given in Figure 4 and their main characteristics are listed in Table 1.

The catchments are located all over the Hérault region and were chosen to be different from each other considering their physiographic descriptors. Three pairs of catchments (C3 and C4 which are nested catchments, C2 and C5, C10 and C11) are very close from each other in order to investigate their hydrological behaviour under the same rainfall conditions.

The climate is Mediterranean type, exhibiting a bimodal temporal distribution with two major rainy periods (one in Spring and the other in Autumn) and high intensity, short duration storms. Two gauged catchments are among this catchment network. The experimental Roujan catchment (0.91 km^2^, catchment G1), monitored by the French National Institute for Agricultural Research (INRA) since 1992, was used to diagnose the main hydrological processes in this region due to it representative position. The Lez catchment, which is larger than the others (116 km^2^) and has permanent flows, is monitored by the DREAL Languedoc-Roussillon Institute. The annual rainfall varies between 500 and 1,400 mm, the mean annual temperature is approximately 14 °C, while the mean annual Penman evapotranspiration is about 1,090 mm. Runoff genesis is dominated by Hortonian overland flows occurring when rainfall intensities exceed soil infiltration capacity [35].

At the small catchment scale, this implies that streams are ephemeral and catchment outflows correspond to runoff events of low frequency (around six per year) but high magnitude and short duration [16].

## 3. Results

The proposed experimental strategy aimed to estimate rainfall and stream flow temporal patterns at the catchment scale. Tests have been conducted in the laboratory and under field conditions in order to improve the monitoring setting. This section shows how hydrological indicators were derived from data collected with the capacitive sensors.

### 3.1. Laboratory Tests

**Figure 5.**
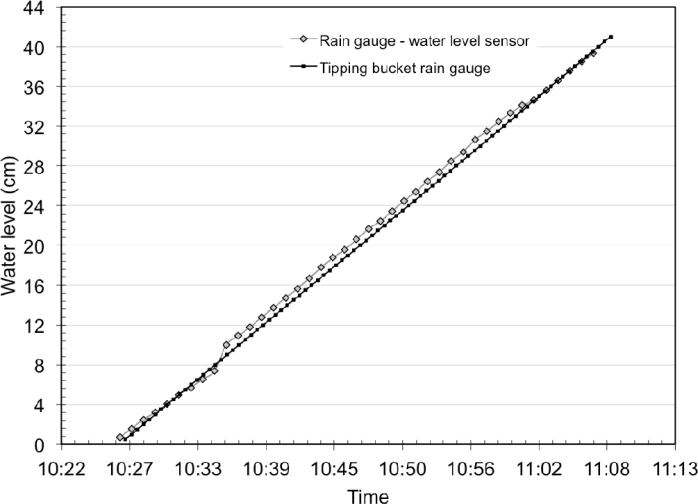
Rain gauge test in laboratory. Comparison between the rain gauge using the capacitive sensor and a reference tipping bucket rain gauge.

The sensor was preliminary calibrated, *i.e.*, the linear relation between output frequencies and water levels was estimated. Secondly the sensor capacity and accuracy to measure rainfall amount and intensity was determined. A tipping bucket rain gauge was taken as the reference device. Both devices received the same rainfall event: 41 mm at a constant intensity of 11.5 mm per 10 min. Figure 5 shows the obtained cumulated rainfall amount during a test period of thirty minutes. The results highlight that the rain gauge using the capacitive sensor gives accurate results. The error on the total recorded rainfall amount is 1.60%.

While extensive laboratory testing was completed, the sensor needed to be tested in the field condition to determine its relevance.

### 3.2. Field Tests

Field testing was carried out considering two rivers which have agricultural catchments, both located in the Mediterranean Hérault region, South France: the Lez and the Roujan catchments. The Lez catchment is monitored by the DREAL Languedoc-Roussillon Institute with a hydrostatic pressure transmitter limnimeter. The gauged channel cross-section is about 10 m wide and 2 m deep and water level ranges between 0.50 m during low-flow period to 2 m height (expressed in limnimetric relative height) during the high-flow period. The Roujan catchment is smaller (0.96 km^2^) with intermittent flows and is monitored by the French National Institute for Agricultural Research (INRA) with a Venturi channel and a meteorological station. The gauged channel cross-section is 1.50 m wide and 1 m deep and water levels range between 0 to 1 m during a runoff event.

**Figure 6.**
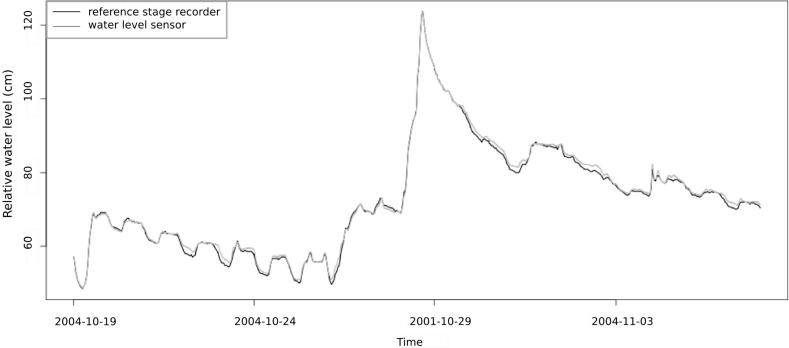
Stage recorder field test carried out at the Lez catchment. Comparison, over a period of sixteen days with a 3-minute data acquisition step, between the stage recorder using the capacitive sensor and a reference stage recorder (DREAL Institute).

**Figure 7.**
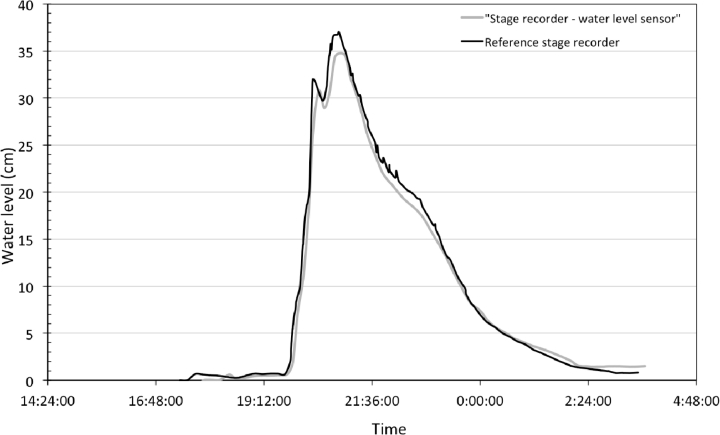
Stage recorder field test carried out at the Roujan catchment. Comparison, over a period of two days with a 1-minute data acquisition step, between the stage recorder using the capacitive sensor and a reference stage recorder (INRA Institute).

**Figure 8.**
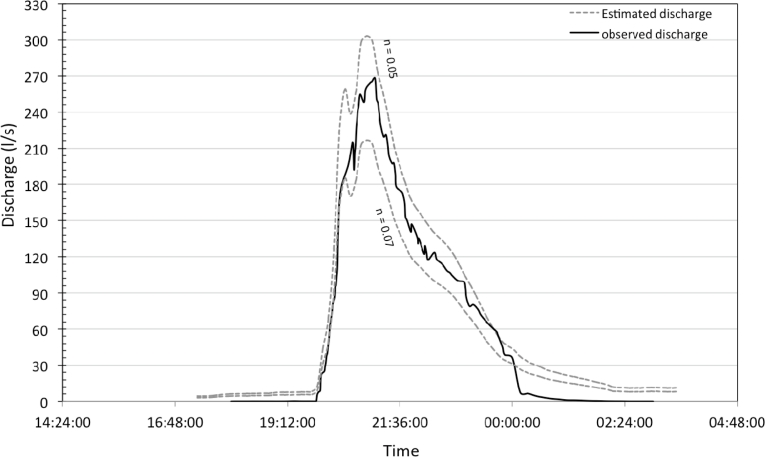
Stage recorder field test carried out at the Roujan catchment. Comparison, over a period of two days with a 1-minute data acquisition step, between discharges estimated using the capacitive sensor and discharges measured with a reference Venturi channel (INRA Institute).

The water level sensor was tested as a stage recorder by comparing the water level measurements to the observations given by the reference stage recorders from DREAL and INRA Institutes. Figures 6 and 7 show the results during two different events and different flow conditions, intermittent and permanent. In both cases the streamflow dynamics were well reproduced, especially during rising limb compared to falling limb where hysteresis phenomena can occur due to the remaining moisture in capacitive wires. The time precision of obtained limnigrams is related to sensor time sampling rate (1 min) with no trend was observed. Water level was estimated as unbiased with an accuracy of 0.7 cm for one standard deviation. According to those results, water level to water discharge conversion was realized using Manning’s *n* roughness coefficients (min. and max.) for intermittent flow and grass cover. Figure 8 shows the comparison between the observed and the estimated discharge at Roujan’s outlet. It highlights that discharges are well estimated as the envelope curve includes the observed discharge. Peak discharges estimated from water level range between 210 to 300 L/s and the observed peak discharge is 270 L/s. This relative error of −22% and +10%, respectively, is within the usual confidence band of discharge measurements during peak flows using rating curves [36,37]. The relative error on runoff volume is acceptable too, ranging between 7.5 (for n = 0.05) and 29.5% (for n = 0.07). If peak discharge and runoff volume are estimated with an associated discharge, the water level sensor allows to calculate exactness (unbiased) the time to peak characterizing catchment reactivity to a rainfall event.

**Figure 9.**
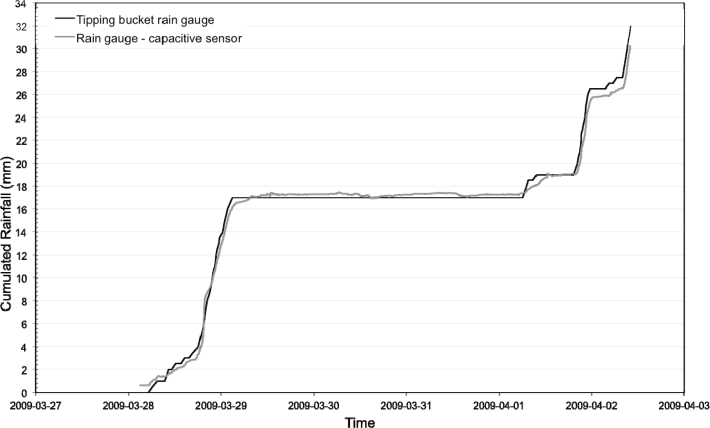
Rain gauge field test carried out at the Roujan catchment. Comparison between the rain gauge using the capacitive sensor and a reference tipping bucket rain gauge (INRA Institute).

The sensor was also tested as a rain gauge by comparing cumulated rainfall amount estimated using the capacitive sensor to observations collected with the reference tipping bucket rain gauge of the INRA. Figure 9 shows the results for a 5 days period. Note the rain gauge gives accurate results over the test period: the two curves fit well, including during the period of 29/03/09 and 01/04/09 where no rainfall occurred. Moreover, the error on total rainfall amount is low, 5.40%, which represents a difference of 2 mm between observed and estimated data.

All experimental tests provided accurate results. The monitoring setting has thus been validated under laboratory and field conditions at different scales by comparing measurements with those obtained with reference devices (tipping bucket rain gauge, hydrostatic pressure transmitter, Venturi channel): rainfall intensities and dynamic responses were accurately reproduced and discharges were estimated with an uncertainty usually accepted in hydrology. On the basis of these validation results, the monitoring setting has been deployed to characterize hydrological behaviour of eleven small headwater agricultural catchments in Mediterranean climate.

### 3.3. Multi-Sites Hydrological Characterization

**Figure 10.**
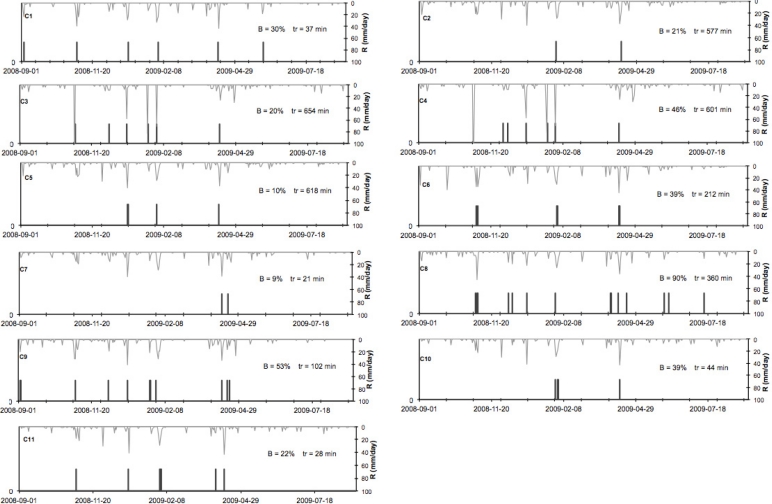
Annual stream flow time series. *B* is the frequency of occurrence of a runoff event and *tr* denotes the mean rising times, both indicators are calculated for the eleven catchments.

The annual stream flow time series and *B* were calculated for each of the eleven catchments and shown with mean rising times in Figure 10.

The results show a high variability in the catchment responses. *B* values range between 9% and 90% meaning that runoff could appear at the catchment outlet less than one rainfall event on ten (C7) or near to all considered rainfall event. Differences are particularly interesting in the couple of catchments (C2, C5 and C10, C11) which have been exposed to the same rainfall characteristics and where *B* could be twice higher in one catchment than the other.

Minimum value of mean rising time is about 37 min, while the maximum value is about 654 min (C3). The rising time value of a catchment is not so easy to pre-determine for small catchments because a lot of factors could impact its value: drainage area, topographic variations, drainage density and internal hydrological connectivity. Therefore, it is crucial to be able to estimate it by measurement. The proposed soft monitoring approach allows to this estimation. Such variability gives promising perspectives in understanding the reasons how catchments could react to a rainfall event and offers important insight for catchment agricultural water management.

### 3.2. Water Budget at Catchment Scale

**Figure 11.**
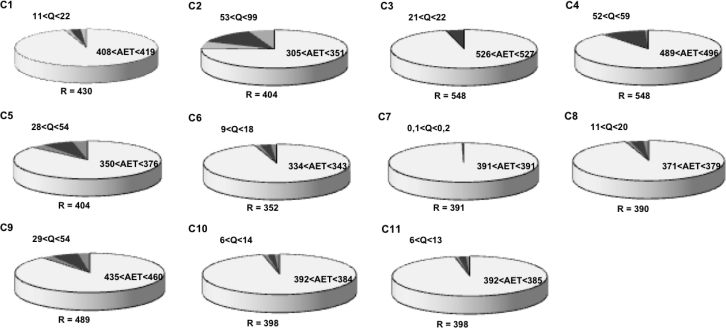
Water budget estimated on the eleven studied catchments for the hydrological year 2008–2009 (*R*, the rainfall amount, *Q*, the catchment runoff and *AET*, the actual evapotranspiration are given as water depth in mm).

Figure 11 shows the water budget estimated on the eleven studied catchments for the hydrological year 2008–2009. The annual rainfall depths range between 352 and 548 mm showing variations in the studied spatial extent (40 km by 30 km) according a North-South gradient well known in the region. The actual evapotranspiration is clearly lower than the mean inter-annual potential evapotranspiration (1,000 to 1,500 mm). These result emphases the water stress encountered in Mediterranean regions. Catchment runoff is much less than actual evapotranspiration and highly variable from one catchment to another (near 0 for C7 and between 53 and 99 for C2). Differences between catchments are high compared to the errors made during the discharge estimation step. These differences showed that rainfall may remain inside the catchment limits (in C2 case for example) or leave these limits by surface runoff at the catchment outlet. In this late case, the water can wash off pollutants spreading on agricultural area or can be part of a problematic flooding downstream. Comparison of Figures 10 and 11 show that annual catchment runoff may be due to few flood events: at C2, the higher annual catchment runoff is explained by only four events (*B* has one of the lower value of 10%). However this fact cannot be generalized because we show also catchments on which runoff and *B* were high (C4), runoff was low and B high (C8).

These results highlight the potential offered by the experimental strategy in estimating water balance. This estimation is an actual challenge in many ungauged catchments in developing countries where data are lacking as well in other parts where long term monitoring are needed to evaluate the impacts of land use and climate change.

## 4. Conclusions

The proposed experimental hydrological strategy for agricultural catchments monitoring is based on innovative soft capacitive sensors designed to be able to record both rainfall intensities and water levels with an accuracy which is still relevant for catchment behavioural inference. Low cost and energy consumption optimization of the proposed sensor allows one to multiply it in space and to study a collection of catchments. Moreover, its design and properties (handy, easy to install and uninstall, low-cost, *etc.*) make the proposed sensor mobile and soft, which allow one to simultaneously monitor several catchments with high temporal resolution.

This study demonstrated that the proposed experimental monitoring setting was fully operational in characterizing agricultural catchments, even under difficult conditions where rains and flows are short and intense. It gave useful results in management perspectives involving pollutant transport, flooding event or global water balance equilibrium. High contrasts in small agricultural catchment hydrological behaviours were revealed. Finally, hydrological indicators could be elaborated for fast diagnosis of a lot of catchments.

On a more technological point of view, there is probably room to increase the relevance of the proposed sensor for agricultural catchment monitoring. For instance, future sensor developments could involve wireless communication between sensors for dense and more secured nested catchment monitoring. Future works could also couple the proposed sensor to an equivalent low-cost flow velocity sensor to measure discharge even in reaches with singularities or where Manning’s law cannot be applied anymore.

## Figures and Tables

**Table 1.** Catchment characteristics.

Catchments	Location	Area (km^2^)	Geology (Main type)	Land use (Main type)
C1	43°28′29″N, 03°14′40″E	0.33	Molasse and Limestone	Vineyard
C2	43°23′43″N, 03°17′40″E	0.60	Limestone	Row-crops
C3	43°28′29″N, 02°58′11″E	0.10	Schist	Forest
C4	43°28′17″N, 02°58′43″E	0.50	Schist	Vineyard
C5	43°23′04″N, 03°17′03″E	0.20	Limestone	Vineyard
C6	43°25′37″N, 03°22′42″E	0.34	Molasse	Vineyard
C7	43°21′51″N, 03°08′07″E	0.20	Molasse	Vineyard
C8	43°25′58″N, 03°29′11″E	0.40	Limestone	Vineyard
C9	43°22′27″N, 03°02′11″E	0.40	Limestone	Vineyard
C10	43°16′54″N, 03°09′34″E	0.40	Limestone	Scrubland
C11	43°16′49″N, 03°09′48″E	0.30	Limestone	Scrubland
G1	43°29′46″N, 03°19′26″E	0.91	Limestone	Vineyard
G2	43°37′07″N, 03°53′48″E	116	Limestone	Forest/urban
